# Sex estimation with convolutional neural networks using the patella magnetic resonance image slices

**DOI:** 10.1007/s12024-025-00943-7

**Published:** 2025-02-19

**Authors:** Nevin Cavlak, Gökalp Çınarer, Mustafa Fatih Erkoç, Kazım Kılıç

**Affiliations:** 1 Department of Forensic Medicine, Faculty of Medicine, Yozgat Bozok University, Yozgat, Türkiye; 2 Department of Computer Engineering, Faculty of Engineering-Architecture, Yozgat Bozok University, Yozgat, 66900 Türkiye; 3 Department of Radiology, Faculty of Medicine, Yozgat Bozok University, Yozgat, Türkiye; 4 Department of Computer Technologies, Yozgat Vocational School, Yozgat Bozok University, Yozgat, Türkiye

**Keywords:** Sex estimation, Deep learning, Patella, Forensic anthropology, Convolutional neural networks

## Abstract

Conducting sex estimation based on bones through morphometric methods increases the need for automatic image analyses, as doing so requires experienced staff and is a time-consuming process. In this study, sex estimation was performed with the EfficientNetB3, MobileNetV2, Visual Geometry Group 16 (VGG16), ResNet50, and DenseNet121 architectures on patellar magnetic resonance images via a developed model. Within the scope of the study, 6710 magnetic resonance sagittal patella image slices of 696 patients (293 males and 403 females) were obtained. The performance of artificial intelligence algorithms was examined through deep learning architectures and the developed classification model. Considering the performance evaluation criteria, the best accuracy result of 88.88% was obtained with the ResNet50 model. In addition, the proposed model was among the best-performing models with an accuracy of 85.70%. When all these results were examined, it was concluded that positive sex estimation results could be obtained from patella magnetic resonance image (MRI) slices without the use of the morphometric method.

## Introduction

In mass disaster situations, identification processes implemented in the presence of highly decomposed corpses or skeletal human remains form the basis of forensic and archaeological studies [1].

The negative aspects of the DNA- and fingerprint-based methods, which are effectively used in identification tasks, are the unavailability of appropriate tissue samples, the fact that these methods are costly to perform in cases involving more than one person whose remains exhibit impaired integrity, and the lack of a database for comparing the data to be obtained. Therefore, biological bone profiling continues to be an important method. Sex estimation is one of the first steps that must be taken since the biological profiles of people affect other descriptors and can eliminate a significant portion of the missing persons list [2–5].

While the probability of obtaining positive identification results increases in cases where most of the bones in a person’s body are available, factors such as environmental conditions, animal activity, cases in which bodies are disintegrated and dumped in different areas, and the formation of artifacts during excavation processes limit the ability to reach the head, pelvis, and long bones, which are frequently used in sex estimation tasks [6, 7]. These limitations increase the need for sex estimation methods based on bones such as the patella, which are relatively resistant to thanatological effects [6, 8].

The patella is a large sesamoid bone with the form of a roughly inverted triangle that is located in the quadriceps tendon [9]. The patella, which has a cartilage structure at week 14 of the gestation process, can be radiologically observed at ages 2–3 as primary ossification center foci, but it is often found at ages 5–6 [10, 11], and the ossification process of the patella continues until puberty [12].

In addition to morphological and metric measurement studies conducted on dry bones for sex estimation purposes, studies involving radiological images are becoming increasingly common today [13]. Radiological bone images of living people contribute to ensuring appropriate sample sizes in sex estimation tasks and increasing the reliability of the obtained results. However, performing metric measurements over radiological images has several disadvantages, such as being dependent on the attention of the person making the measurement, the use of a limited number of parameters, and the lack of standard methods [14].

Deep learning algorithms, which offer better results than manual feature extraction systems do, have recently been increasingly used in the field of medicine [15]. Convolutional neural networks (CNNs) are deep learning techniques that are widely used in image processing scenarios because of their ability to comprehend complex features and maintain spatial connections between features [16]. A CNN has an architecture consisting of a series of convolution, pooling, and connected layers, and it can automatically identify patterns beyond those determined through human visual perception by using relevant layers [17]. Studies in the field of forensic anthropology have used CNN models as mediators for determining sex from different bone images [14, 18–20]. However, to our knowledge, no study has been conducted with a CNN on the patellar bone. In this study, we aimed to investigate the effectiveness of patella magnetic resonance image (MRI) slices in sex estimation tasks via CNN models, develop a fast and repeatable CNN model, and compare its accuracy rates to those of the existing models.

## Materials and methods

### Dataset

**Fig. 1:**
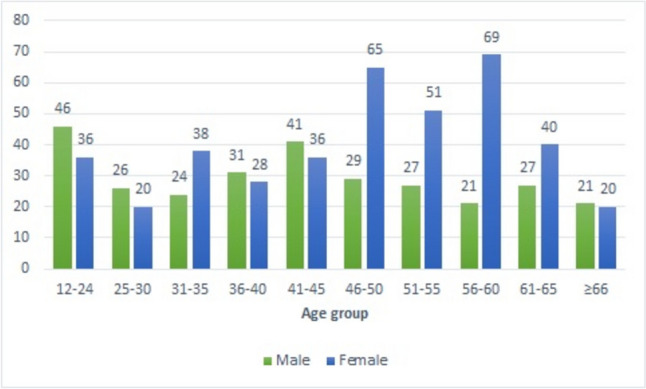
Distribution of cases by sex and age groups

A large quantity and various types of patella images are needed to train a CNN model. For the training process of the model to be developed, sagittal 1.5-T MR knee images obtained from the radiology department of a university hospital, which were taken for different reasons, were used. In line with studies stating that there are no significant differences between the right and left patellae [21–23], 6710 T1-weighted (T1W) MR sagittal patella image slices of 696 patients (293 males and 403 females) were included in the study. The images included patients between the ages of 12–80 years (males: 12–75 years, females: 14–80 years) who were evaluated at the hospital. Data were analyzed using Statistical Package for Social Sciences (SPSS) version 22 (IBM Corp., Armonk, NY). Pearson chi-square test was applied, crosstabs with a z-test with Bonferroni correction to analyze categorical variables between different groups, and the significance ratio was determined as *p* < 0.05. After Bonferroni correction, no significant difference was observed between sex groups except for the 56–60 age group (χ2 (9) = 38.537 *p* < 0.001) (Fig. 1).

### Radiologic assessment

**Fig. 2:**
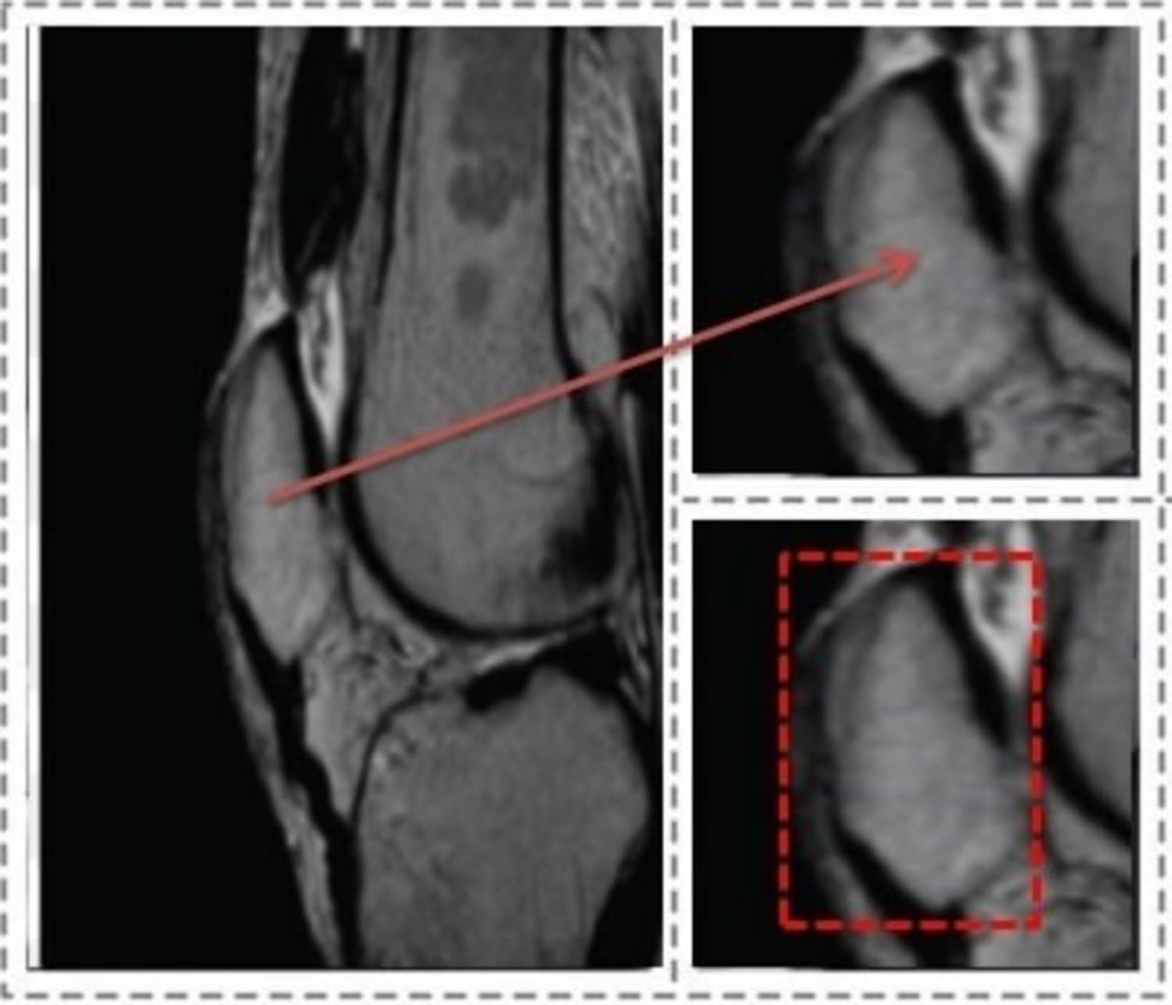
The patella MR image dataset

All patients were examined via a 1.5-T MRI system (Philips Ingenia, Philips, Germany) with an 8-channel sensitivity encoding (SENSE) extremity coil. The imaging parameters for the T1W sagittal slices were 3810 ms/114 ms/1 (repetition time [TR]/echo time [TE]/ average number of signals [NSA]), a 150-degree flip angle, a 259 × 118 matrix, an 800-cm field of view (FOV), a 130-kHz bandwidth, an echo train length of 13, and a 4-mm slice thickness, with an acquisition time of 4 min, 26 s. All of the images were labelled by a senior radiologist who was blinded to the clinical statuses of the patients. Images with artifacts, bone fractures, operations, and malignant findings were excluded from the study. A sample patella image from the dataset is shown in Fig. 2.

### Ethics

The Clinical Research Ethics Committee approved the study. All aspects of the study were performed in accordance with the ethical standards of the 1964 Declaration of Helsinki and its later amendments or comparable ethical standards. Since this study performed retrospective sampling from a preexisting image database and the identity information of the images was removed, the Ethics Committee did not require patient consent.

### Data preprocessing

In recent years, deep neural networks have been successfully utilized in the field of medical image processing to classify radiological images. [21]. The most popular architectures used for this purpose are CNN models. A CNN model minimizes the required data preprocessing work and learns attributes from enormous quantities of data via convolution and pooling functions. Numerous studies have investigated the diagnostic use of CNNs in radiological imaging tasks [22–24]. CNN architectures are the most popular deep learning architectures; they are widely used in image analysis scenarios and have produced successful results. The popular CNN architectures used in this study for image classification purposes were EfficientNetB3, MobileNetV2, Visual Geometry Group 16 (VGG16), ResNet50, and DenseNet121. These architectures were trained on more than two million images in the ImageNet competition and have proven their success.

### Training procedure

A Rectified Linear Unit (ReLU) was used for activation in convolution layers during the training phase. Adamax optimizer was used for model optimization. Learning rate was determined as 0.001. When valid loss was not reduced during the training phase, learning rate was reduced by 0.50. The number of training epochs was set to 100 and the batch size was set to 64. For training and testing of the proposed architecture, the data set was divided into 70% training, 15% validation and 15% test.

### EfficientNetB3

EfficientNet is a deep learning model that requires less computational power to achieve higher accuracy rates than those of other methods. It was developed by Google Brain [25]. EfficientNet is a scalable deep learning model that scales feature maps in a regular manner to achieve better feature extraction effects by optimizing features at every level of the network. EfficientNet scales the size and depth of the constructed model via a method called "compound scaling". This method integrates the width, depth, and resolution of the network to create a scalable model. It also uses an activation function called "swish". Swish is a nonlinear function such as a ReLU, but it provides a smoother transition.

### VGG16

VGG16 is a type of deep learning model known as a CNN [26]. This model is used in visual recognition, object classification, object detection, and other visual processing tasks. The VGG16 model accepts RGB images as its inputs and scales them to 224 × 224. The first layer of the model processes the input image through 64 different filters and generates a 64-channel image. Each of these channels represents a feature map. The model then adds two more layers, further processes the feature maps, and obtains higher-level features. The final layers are fully connected layers that are employed for the classification process. These layers create a vector out of the obtained feature maps, and this vector is sent to a softmax classifier used for classification. Finally, the VGG16 model is trained on the ImageNet dataset.

### ResNet50

The residual network (ResNet) is a deep learning model that was developed by Microsoft researchers in 2015 [27]. ResNet aims to solve the performance degradation problem that occurs as networks become deeper. To avoid this performance degradation, it focuses on eliminating the problems associated with the backward propagation of the network in deeper layers. ResNet uses an innovative connection technique named "residual connections" to eliminate the degradation problem. Thus, directly adding the outputs of the layers to the input of the next layer ensures that the properties of the previous layers are preserved in the subsequent layer [27]. ResNet also stands out due to its model variants with different depths (18, 34, 50, 101, 152, etc.). These models have different numbers of layers, and each has different performance and complexity levels [28]. The ResNet50 model was trained on the ImageNet dataset and consists of 50 layers. This model has a more complex structure in which "bottleneck" layers are used. Bottleneck layers are preferred for obtaining a lighter and faster model.

### DenseNet121

DenseNet121 is a CNN model that was developed in 2017 [29]. DenseNet is known for its features called “dense connections.” These links are created by using the output of each layer as the input of the next layer. Thus, the properties of the previous layers are also used by the subsequent layers. DenseNet121 is a 121-layer model. It accepts 224 × 224 RGB images as its inputs. The model consists of several dense blocks, normalization layers, and pooling layers. Dense blocks are clusters of layers in which dense connections are used, and each block transforms the properties of the previous layers into new features. Normalization layers are used to prevent overfitting. Pooling layers reduce the sizes of feature maps and provide local translation invariance for the features. DenseNet121 also uses “bottleneck” layers. These layers help with learning smaller feature maps and reducing the number of model parameters.

### MobileNetV2

MobileNetV2 is an artificial neural network architecture that was developed by Google and designed specifically for use in applications on mobile devices. Compared with its previous version, MobileNetV2 was designed to provide better performance, a lower error rate, faster operations, and less memory consumption [30]. Utilizing a technique called kernel learning, MobileNetV2 can better learn the weights and parameters of the network. It also uses a structure called "depthwise separable convolution". In this structure, calculations are performed via one depthwise convolution and one pointwise convolution.

### Image preprocessing

Image processing is generally a preferred practice when classifying data related to some special information obtained by different methods applied to an image. In the first stage of the proposed method, the relevant region (the patella) was separated from the MR knee images via segmentation techniques. Since the obtained patella images may have been of different sizes, they needed pretreatment. For this reason, all the images were reduced to a uniform size. The images were integrated into the deep neural network model that was created accordingly. This deep learning model was developed for performing feature extraction and classification and estimation on images. In addition, image sharpening, which is a type of image filtering, can be used to enhance the clarity of images by reducing noise in radiological images. [31]. In this study, such procedures were not applied to prevent deformation of the original images. The CNN architectures used for image processing accept images with dimensions of approximately 224 × 224 and 300 × 300. For this reason, the performance of the proposed model was also enhanced within the scope of the study by providing a fast deep learning architecture with high accuracy that can process and analyze small patella images.

### Architecture of the model

**Fig. 3:**
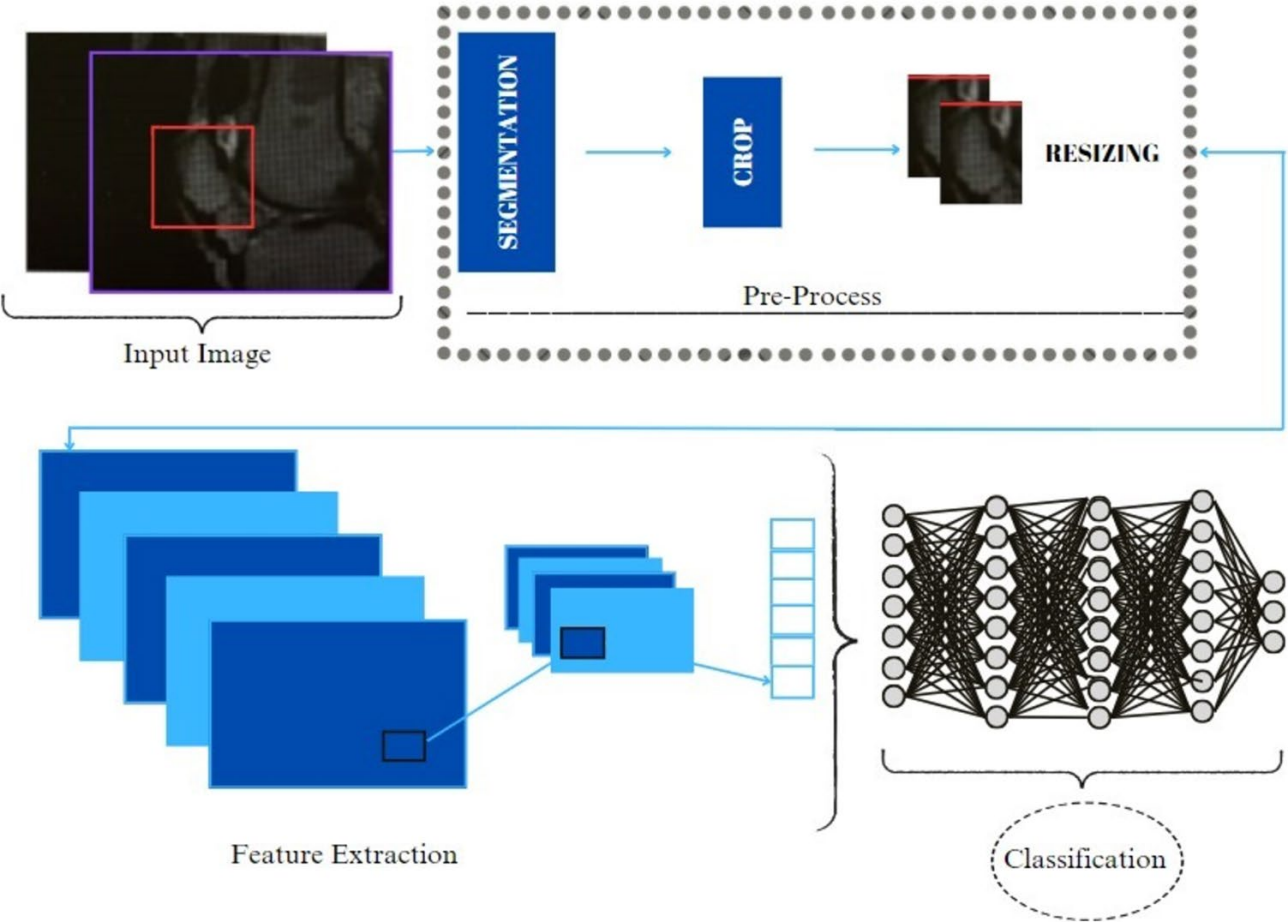
Flow diagram of the proposed model

CNNs are the most popular deep learning architectures that accept images as their inputs. A CNN architecture is generally formed with fully connected, convolution, pooling, and activation layers. In the last stage of the proposed method, the attributes of the patella images were extracted and classified via a CNN. The general architecture of the developed method is presented in Fig. 3.

In this study, different-sized patella images were reduced to an average standard size, and a CNN architecture, which obtained the most successful analysis results for these images, was utilized. The layer structure and basic architecture of the model are presented in detail in Table 1.

**Table 1:** Layer structure and architecture of the model

Layer (type)	Output Shape	Parameters
conv2d (Conv2D)	(None, 64, 64, 64)	4864
conv2d_1 (Conv2D)	(None, 64, 64, 64)	102,464
max_pooling2d (MaxPooling2D)	(None, 32, 32, 64)	0
dropout (Dropout)	(None, 32, 32, 64)	0
conv2d_2 (Conv2D)	(None, 32, 32, 128)	73,856
conv2d_3 (Conv2D)	(None, 32, 32, 128)	147,584
max_pooling2d_1 (MaxPooling 2D)	(None, 16, 16, 128)	0
dropout_1 (Dropout)	(None, 16, 16, 128)	0
flatten (Flatten)	(None, 32,768)	0
dense (Dense)	(None, 256)	838,884
dropout_2 (Dropout)	(None, 256)	0
dense_1 (Dense)	(None, 2)	514

### Performance evaluations

Evaluating the performance of algorithms is important for measuring their functionality and accuracy. Basic success criteria for algorithms, such as the accuracy, precision, and F1 score metrics, are values that should be known at the classification stage. Performance evaluations also facilitate the testing of algorithms on different datasets to measure their success. Thus, evaluating the performance of algorithms is a crucial step toward better decision-making processes, efficient resource usage, and better results. Table 2 presents the confusion matrix used to calculate these metrics. The basic formulas used to calculate the performance of the proposed algorithm according to the confusion matrix are as follows:

**Table 2:** Confusion matrix

Estimated values	Actual Values	
	Positive	Negative
Positive	True Positife (TP)	False Positive (FP)
Negative	False Negative (FN)	True Negative (TN)

$$\text{Accuracy }= \frac{TP+TN}{TP+TN+FP+FN}$$

$$\text{Sensitivity }(\text{Recall}) = \frac{TP}{TP+FN}$$

$$\text{Specificity }= \frac{TN}{FP+TN}$$

$$\text{Precision }= \frac{TP}{TP+FP}$$

$$\text{F}1\text{ Score }= \frac{2XPrecisonxRecall}{Precison+Recall}$$

## Results

One of the major challenges encountered in deep learning tasks is excessive fitting, which is caused by the use of many model parameters and the complexity of the employed regularization techniques. Therefore, to enable model generalization, the input data are typically divided into three clusters: a training set for hyperparameter optimization, a validation set for overfitting control, and a test set for estimation. The hyperparameters of the model developed in this study were also specifically optimized according to the given dataset. Unlike the classic measurement-based classification models found in the literature, a special model architecture based on deep learning was used to obtain the results in this study. The performance-related results of the model are presented in Table 3. The proposed CNN model was able to accurately classify patella images with precision, F1 score, and recall rates of 85.71%, 85.67%, and 85.70%, respectively. According to the results obtained within the scope of the study, the proposed model achieved high performance, with an overall accuracy of 85.70%. The classification results were highly accurate even when the parameters and layer structure of the CNN model were determined with a new model, which was also a contribution of the study. In addition, despite the high similarities among the patella images, the performance similarities between the proposed model and the other four classification algorithms included in the study support the success of another important classification process in this field.

**Table 3:** Performance of the model

	Precision	Recall	F1-score	Support
0 (Female)	0.8544	0.8887	0.8712	548
1 (Male)	0.8604	0.8192	0.8393	459
**Accuracy**			0.8570	1007
**Macro Avg**	0.8574	0.8539	0.8552	1007
**Weighted Avg**	0.8571	0.8570	0.8567	1007

**Fig. 4:**
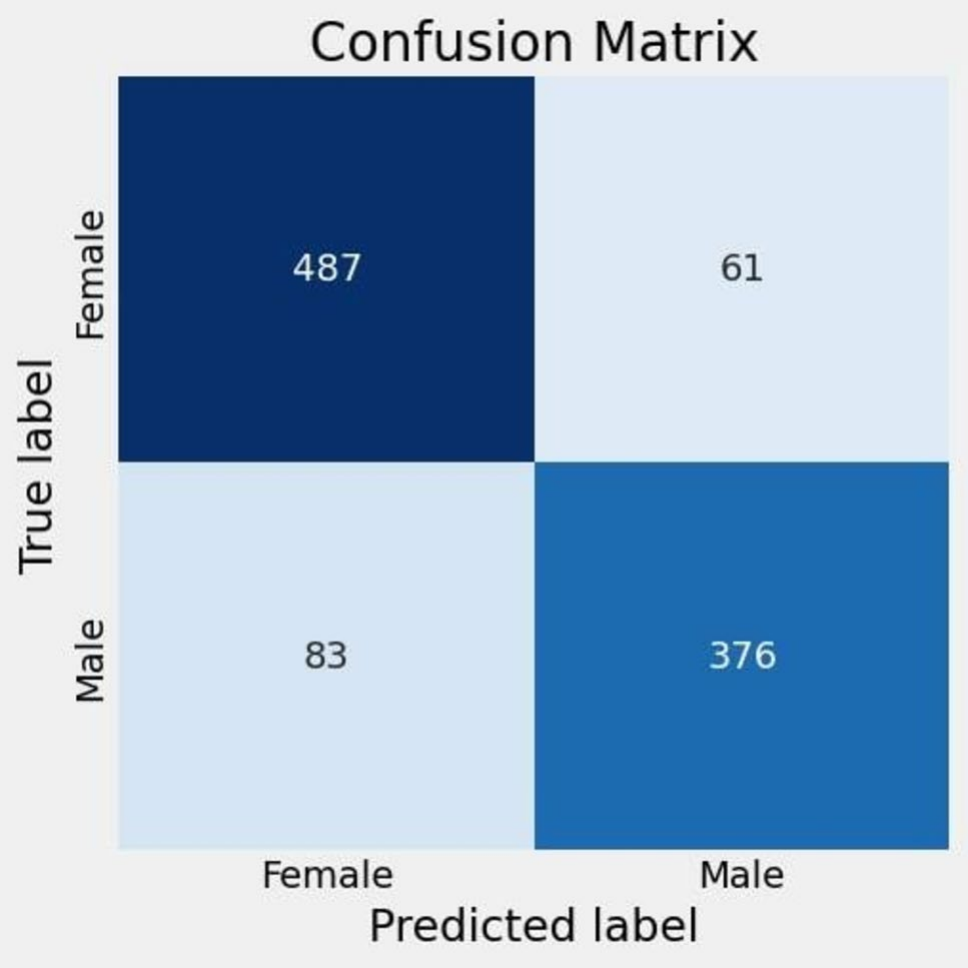
Confusion matrix results of the models

**Fig. 5:**
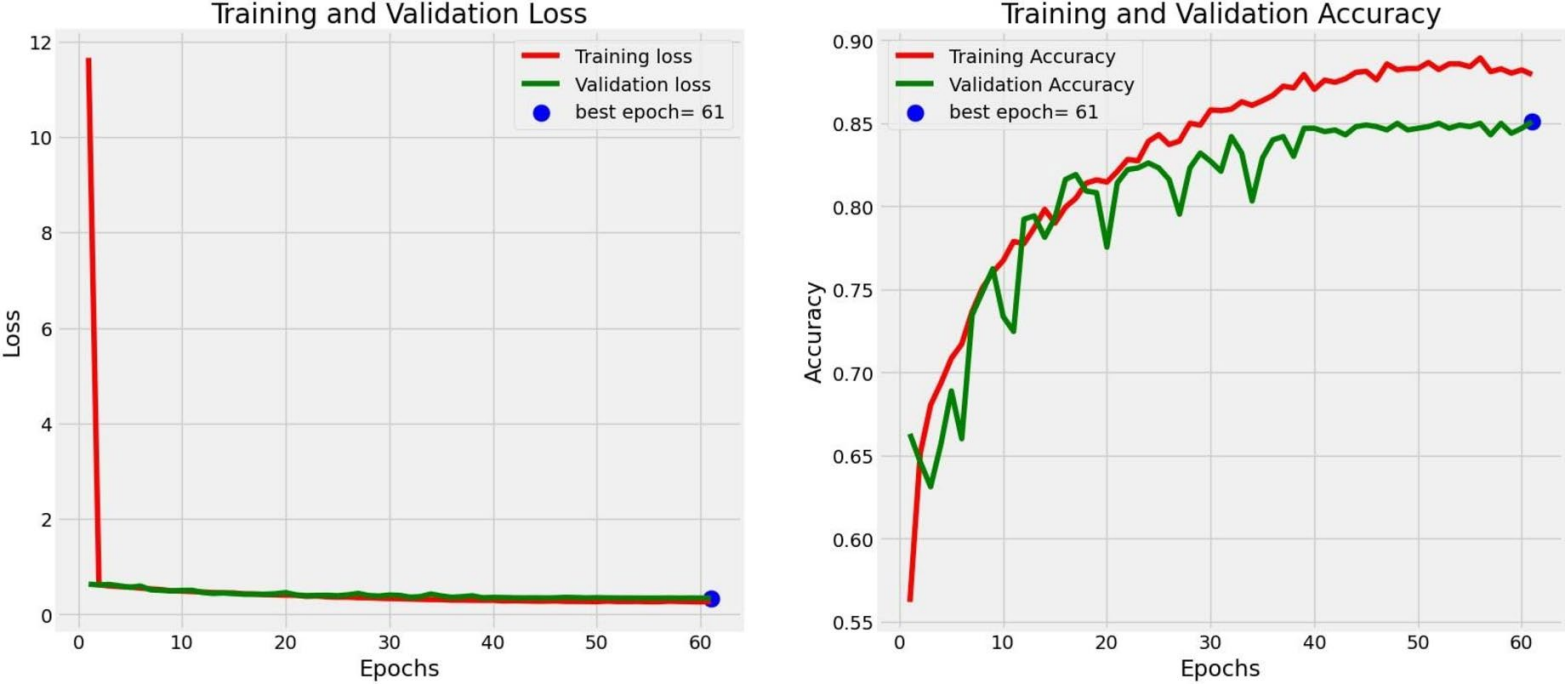
Loss and accuracy performance indicators of the models

The extraction and classification results obtained for the deep features of the patella images through the CNN proposed in this study are presented in the confusion matrix provided in Fig. 4. The loss values and accuracy values of the proposed model are shown graphically in Fig. 5. Here, the prediction accuracy and loss function produced by the CNN model for patella images are shown. The accuracy and loss values were obtained for each period. Each period underwent a cycle to update its weight during the total data training process. The loss values determined the degree of the model response to each iterative optimization step. In other words, to obtain the most appropriate weights for the model, the loss values trained the total errors determined for each image. When the training and test curves were examined in detail, the proposed model displayed very consistent performance in terms of classifying the patella images. Incorrectly setting parameters such as the learning speed in a network can cause the associated model to be overadjusted and unable to reach convergence. This situation did not occur in the proposed model, and the loss value decreased consistently in the training and validation groups. In addition, the accuracies of the training and validation values similarly increased as the number of epochs increased. Moreover, the best result in both tables were reached after 61 epochs.

**Fig. 6:**
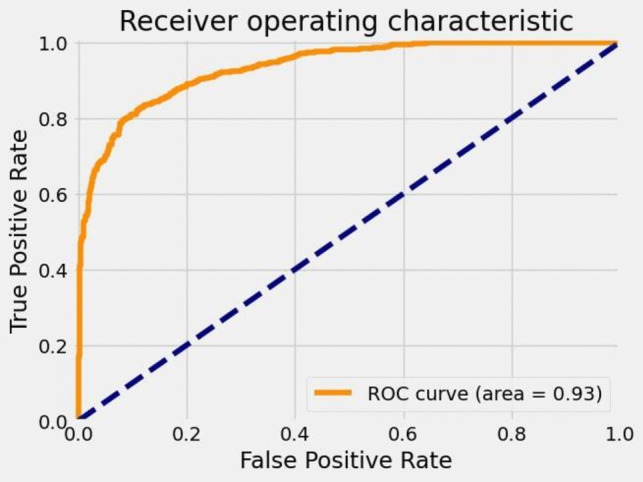
The ROC curve of the proposed model

The receiver operating characteristic (ROC) curve of the model proposed in this study is shown in Fig. 6. When the ROC curve was examined, it was determined that the model had a very high performance level of 93%. When the ROC curve was close to 1.0, the performance of the model was also very high.

Table 4 summarizes the accuracy, precision, sensitivity, specificity, and area under the ROC curve (AUC) values produced for the patella images extracted by the CNN of the proposed model and other architectures; these values were calculated from the corresponding confusion matrices.

**Table 4:** Performance comparison of the models

Model	Accuracy	Precision	Recall	F1-score	AUC score
Proposed	0.8570	0.8571	0.8570	0.8567	0.9341
EfficientNetB3	0.8540	0.8554	0.8540	0.8542	0.9256
VGG16	0.8769	0.8776	0.8769	0.8764	0.9515
ResNet50	0.8888	0.8887	0.8888	0.8888	0.9477
DenseNet121	0.8878	0.8879	0.8878	0.8878	0.9542
MobileNetV2	0.8371	0.8375	0.8371	0.8366	0.9165

When other patella image classification architectures were examined, the ResNet50 architecture obtained the highest result, with 88.88% accuracy. In addition, the precision value of this model was 88.87%. Considering the AUC values of the architectures, the DenseNet121 model had the best performance at 95.42%. The accuracy values of all the models were above 82%, indicating that this type of method is important for performing sex estimation based on patella images. In addition, the accuracy of the proposed model was greater than those of the MobileNetV2 and EfficientNetB3 architectures. This showed that the proposed model was quite successful. The EfficientNetB3 architecture with 85.40% accuracy and MobileNetV2 with 83.71% accuracy were the two models with the lowest accuracy values. When the recall values considered while determining the classification performance of the different models were examined, MobileNetV2 obtained the worst results.

## Discussion

The ability to reach trained personnel during major disasters and prolonged periods of war, housing opportunities for personnel, and situations where the safety of the working environment cannot be ensured are important factors that reveal the need for automatic identification processes. The determination of sex from bones with artificial intelligence methods can contribute to preventing the problems encountered during identification tasks. In this study, we proposed a practicable CNN-based sex estimation method that works with patella MRI slices.

When the existing studies on conducting sex determination based on the patella were examined, the data obtained via radiological image measurements as well as manual measurements implemented over dry bone were generally statistically analyzed via discriminant factor analyses or ROC curves (Table 5). In cases where the measured parameters were singular, the accuracy rates varied between 62.5–91.2%, whereas when the parameters were combined, the accuracy rates ranged from 70–93.3% [2, 3, 6, 32–40]. Traditional machine learning techniques have also been used in patella-based sex estimation studies. In a study in which the patella measurement values obtained from a cadaver (whose accuracy rate was previously calculated as 92.9% via discriminant factor analysis) were evaluated via the artificial neural network (ANN) method, the accuracy rate was calculated as 96.1% [41]. In another study, in a sex estimation process implemented with the feedforward backpropagation neural network (FFBPNN) method, features were automatically obtained from 3D computed tomography (CT) images of the patella; the training accuracy was 96.02%, and the testing accuracy was 93.51% [7]. In a study that evaluated measurements obtained from patella CT images via the decision tree method, the accuracy rates were 98.2% for males and 98.4% in females [42]. Bidmos et al. reported that the accuracy rate of 84.2% obtained by implementing the discriminate analysis method on measurements of dry bone reached 90.77% when the stacking machine learning method was utilized [43].

**Table 5:** Summary of previous studies on sex estimation from patella

Author	Years	Population	Data	Number Male/Female	Age	Variable	Statistical method	Univarite/multivarite Accurate variable (%)
Introna et al. [6]	1998	South Italian	Skeletal Remains	80 40/40	25–80	H,B,T, MAFB,LAFB MAFH,LAFH	DFA	62.5–78.75/76.3–83.8
Kemkes-Grottenthaler [36]	2005	Prehistoric Skeletal Sample	Skeletal Remains	52 26/26		H,B,T, MAFB,LAFB MAFH,LAFH	DFA	71.2–80.8/74.0–84.6
Mahfouz et al. [7]	2007	Northern American	3D CT of Skeletal Remains (*n* = 195) and patients and cadavers (*n* = 33)	228 133/95	16–97	Geometric features, moments, principal axes, and principal component	FCM DFA FFBPNN	83.77 90.3–91.7 Trainin %96.02–99.29 Testing %93.51–90.9
Sakaue et al. [21]	2008	Japanese	Skeletal Remains	283 183/100	14–83	H, B,T, HAF,MAFB,LAFB	DFA	-/84–85
Akhlagi et al. [2]	2010	Iranian	Extracted bone cadavers	113 57/56	20- ≥ 65	H,B,T	DFA	74.3–91.2/ 92.9
Afrianty et al. [41]	2014	Data from the study conducted by Akhlagi Et Al [2]					ANN	96.1
Peckmann et al. [22]	2016	Spanish	Skeletal Remains	106 55/51	22–85	H, B,T, HAF,MAFB,LAFB	DFA	-/75.2–84.8
Aly et al. [35]	2016	Chinese	X-Ray of Clinical Patients	479 255/224	10–20	H,B,T	DFA	65–73/70–73
Michiue et al. [3]	2018	Japanese	3D postmortem CT	220 110/110	18–95	H,T, Patella volume, Total CT attenuation value	ROC	70,9–87.7/85.7–93.3
Peckmann et al. [23]	2018	African American	Skeletal Remains	200 100/100	20–80	H, B,T, HAF,MAFB,LAFB	DFA	-/80–85
Teke-Yaşar et al. [38]	2018	Turkish	MRI of Clinical Patients	220 110/110	19–75	H,B,T	DFA	79–86.5/89
Zhan et al. [39]	2020	Chinese	3D CT of Clinical Patients	300 156/144	18–90	H,B,T, Patella volüme	ROC	73.1–85.7/81.9–91.6
Rahmani et al. [37]	2020	Iranian	MRI of Clinical Patients	161 79/82	30–50	H,B,T	DFA	65.8–85.1/83.2–85.7
Indra et al. [40]	2021	Swiss	Skeletal Remains	234 117/117	20–85	H,B,T	DFA	62.82–82.48/83.76
Öner et al. [42]	2021	Turkish	CT of Clinical Patients	350 219/131	18–91	H,B,T, Patella volume	Decision Tree	Male: 98.2, Female: 98.4
Bidmos et al. [43]	2022	South African	Skeletal Remains	260 130/130	25–79	H, B,T, HAF,MAFB,LAFB	DFA Stacking ML	69.2–81.5/81.9–84.2 90.77

*H*: Height, *B*: Breadth, *T*: Thickness, *MAFH*: Medial Articular Facet Height, *LAFH*: Lateral Articular Facet Height, *MAFB*: Medial Articular Facet Breadth, *LAFB*: Lateral Articular Facet Breadth, *HAF*: Height of Articular Facet, *DFA*: Discriminant Functional Analysis, *ROC*: Receiver Operating Characteristic, *FCM*: Fuzzy C-Means, *FFBPNN*: Feed-Forward Back Propagation Neural Networks, Machine Learning: *ML*

It is understood that sex estimation tasks in patella studies are generally evaluated based on the data obtained from measurements. This situation involves certain disadvantages, such as the need for trained staff to obtain the measurements, the time-consuming aspect of acquiring more than one measurement for each patella, and differences among the utilized parameters. In this context, owing to the high processing, accuracy, and consistency rates of deep learning models, it is highly important to use them for automatically analyzing radiological images. In this study, unlike traditional classification models, the proposed DL model could automatically extract important features obtained from sagittal slice images of patellae and classify them with an accuracy rate of 85.70%. In addition, the experimental results revealed that the proposed model performed well when classifying patella images in terms of their precision, sensitivity, and AUC values. In this study, among the employed models, the highest accuracy of 88.88% was achieved with the ResNet50 model. In this study, the accuracy rates achieved by all the models used to perform sex estimation from patella MRI slices were greater than 80%, which means that the developed method satisfies the acceptability limit of sex estimation [44].

Sex estimation studies have been conducted in the literature through the CNN method with different bones. Nonthasaen et al. [45] attained the highest accuracy of 87.5% with the InceptionResNetV2 CNN model based on hand radiography images. Khazaei et al. [20] used cephalometric radiography images and reached an accuracy rate of 90% with the DenseNet121 CNN architecture. Bewes et al. [18] reached an accuracy rate of 95% in a sex estimation task by training the GoogLeNet architecture with 2D left-side images obtained from 900 3D CT skull images. Studies conducted by implementing CNN methods over some parts of bones are also noteworthy. Li et al. used the ResNet-18 model to assess proximal femur X-ray images [14] and attained accuracy rates of 94.6% for the Chinese Han population and 82.9% for the White Caucasian population. Venema et al. [46] achieved an accuracy rate of 91.03% in a sex determination task through ResNet50, which they accepted as the best model, by using photographic images of the distal posterior of the humerus.

The studies mentioned above were conducted on parts of bones for use in cases where the discovered bones are not whole, but the examined bone parts had morphological integrity. However, in cases where adequate morphogeometric data cannot be obtained from corpses smashed under debris during disasters such as wars and earthquakes as a result of artifacts created by animals, the efficacy of the developed models can remain limited. In a recent study conducted by Quora et al. [47], the practicability of a CNN-based sex estimation method was investigated via peripheral quantitative CT over only one slice of every fourth lumbar vertebra corpus image taken on the coronal plane at the midcoronal level. In that study, the accuracy rate of the best model was 86.4%; when all sagittal slices of the patella included in the MR image were used, a similar accuracy rate was obtained. In the present study, in cases where morphogeometric data could not be obtained and the integrity of the bones was compromised, the sole use of slice images provided an acceptable accuracy rate. In conclusion, the present study is the first to estimate sex from patella MRI slices through the direct application of deep learning algorithms. Furthermore, the use of different CNN models and changing the number of parameters, the layer structures, and the activation functions reduced the incurred calculation costs and increased the performance of the developed architecture. Moreover, such solutions increased the accuracy and calculation rate of the model, and overfitting was prevented through the applied loss prevention system.

### Limitations

The study had certain limitations. The data used in the study included only one population, so the model needs to be verified on data acquired from different populations. Other limitations of the study included its retrospective nature and wide age range. The fact that the employed MRI technique is more costly than tomography and is not accessible under difficult conditions can be seen as another limiting situation encountered in this study. However, considering that CT and MR images can be converted into each other via deep learning methods [48–52], this situation can be overcome over time.

Radiological images obtained from hospitals significantly contribute to sex estimation by providing many samples and data that are related to the current population. Moreover, more reliable accuracy rates can be attained in sex estimation studies when the CNN method and the anthropometric measurements and disease information of individuals, in addition to their radiological images, are used. Further studies can be conducted by presenting the relevant data to be obtained for different populations with mobile, web-based algorithms.

## Conclusion

The model that was developed herein to estimate sex from patella MR images via the CNN method yielded an accuracy rate of 85.70%, whereas the best accuracy rate was achieved with the ResNet50 deep learning algorithm (88.88%). As a result, in cases where the examined bones do not have morphological integrity, the use of MRI slices can provide forensic examiners with valuable information for conducting sex estimation.

### Key Points

1. The use of deep learning methods in sex prediction tasks is becoming increasingly widespread.
2. The tested CNNs demonstrated the feasibility of performing sex estimation based on patella MR slices.
3. While the developed model yielded an accuracy rate of 85.70%, the best accuracy of 88.88% was obtained with the ResNet50 model.
4. MRI bone slices can be used to perform sex estimation in cases where morphometric bone data cannot be obtained.

## Data Availability

All data generated or analysed during this study are included in this published article.
